# Smale horseshoe structure in the firing rate model

**DOI:** 10.1186/1471-2202-14-S1-P152

**Published:** 2013-07-08

**Authors:** Dennis Guang Yang, Yixin Guo

**Affiliations:** 1 Department of Mathematics, Drexel University, Philadelphia, PA 19104, USA

## 

The firing rate model in the form of nonlinear integrodifferential equations can characterize spatiotemporal patterns of a continuum neural field. These patterns are associated with a wide range of neurobiological phenomena, such as persistent activity and propagating waves in neural networks.

To understand the substrates of neural circuitry that supports the localized stationary patterns, we study the existence of multi-bump pulse solutions of an integral equation that is the equilibrium equation of the firing rate model. If the integral coupling function, which describes the spatial connection among the network of neurons, is even and its positive half solves a second order linear ordinary differential equation (ODE), then the multi-bump pulse solutions of the integral equation are homoclinic solutions of a reversible fourth order ODE. It was known previously that the corresponding ODEs are conservative for a class of oscillatory and decaying coupling functions [1]. We show that for the Amari-type firing rate model, which assumes the Heaviside firing rate function and "Mexican hat" coupling functions that decay exponentially, the corresponding ODEs are also conservative. Then, by analyzing the configurations of stable and unstable manifolds (see the figure below) in the corresponding reversible, conservative ODE system, we establish the existence of the Smale horseshoe for an open set of model parameters such as the decay rate of the coupling and the threshold of the Heaviside firing rate function. Consequently, even for the Amari-type firing rate model there are countably many symmetric and asymmetric multi-bump stationary pulse solutions as well as spatially chaotic stationary solutions. Furthermore, the robustness of the Smale horseshoe implies that similar solutions also exist for nonsaturating piecewise-linear firing rate functions with small gains and for smooth firing rate functions that are "close" to the Heaviside or piecewise-linear case.

**Figure 1:**
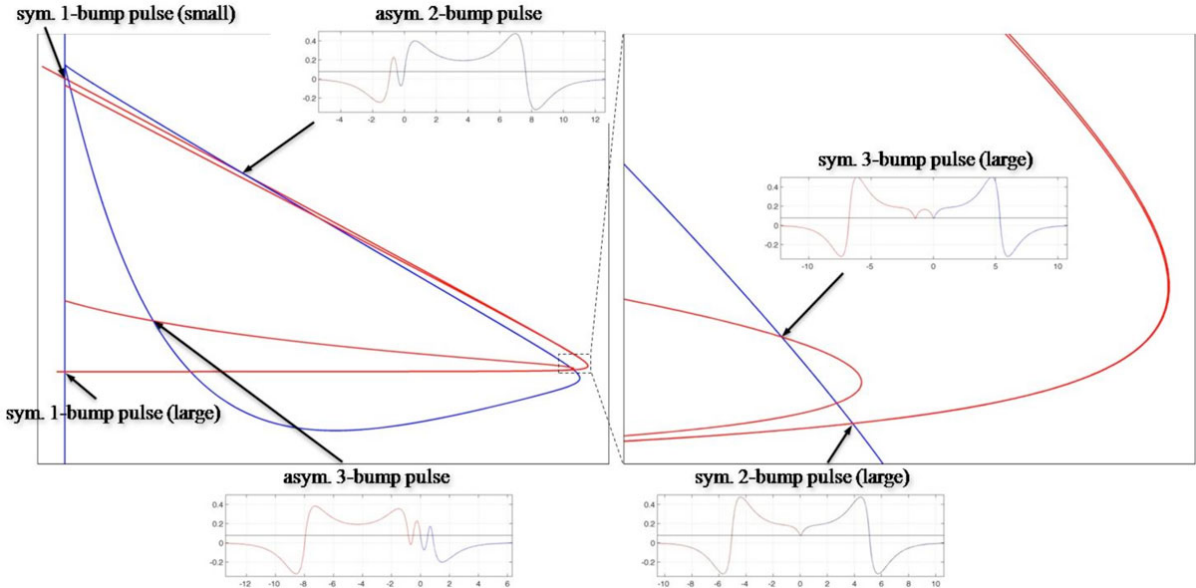
**Multi-bump pulse solutions correspond to intersections between the stable and unstable manifolds of the equilibrium state in the fourth order reversible ODE system**. Since the ODE system is conservative, the intersections between these manifolds can be found by inspecting their projections onto a 2D phase plane. The above figure shows a partial picture of the projection of the stable manifold (blue curves) and the projection of the unstable manifold (red curves). For some of the intersections, the corresponding pulse solutions are plotted. The Smale horseshoe occurs in the full picture of the projections of the stable and unstable manifolds. This implies countably many intersections between the stable and unstable manifolds.
